# Characterization of Newcastle disease virus isolates obtained from outbreak cases in commercial chickens and wild pigeons in Ethiopia

**DOI:** 10.1186/s40064-016-2114-8

**Published:** 2016-04-18

**Authors:** Delesa Damena, Alice Fusaro, Melaku Sombo, Redeat Belaineh, Alireza Heidari, Abera Kebede, Menbere Kidane, Hassen Chaka

**Affiliations:** National Animal Health Diagnostic and Investigation Center, P.O. Box 04, Sebeta, Ethiopia; Research and Innovation Department, Istituto Zooprofilattico Sperimentale delle Venezie, OIE/FAO and National Reference Laboratory for Newcastle Disease and Avian Influenza, OIE Collaborating Center for Diseases at the Human-Animal Interface, Legnaro, Padova Italy

**Keywords:** Newcastle disease virus, Molecular characterization, Chickens, Pigeons, Ethiopia

## Abstract

Newcastle disease (ND), caused by virulent avian paramyxovirus type 1, is one of the most important diseases responsible for devastating outbreaks in poultry flocks in Ethiopia. However, the information about genetic characteristics of the Newcastle disease viruses (NDVs) circulating in commercial chickens and wild birds is scarce. In this study, we characterized isolates obtained from ND suspected outbreaks during 2012–2014 from poultry farms (n = 8) and wild pigeons (n = 4). The NDVs isolated from pathological specimens, through inoculation in embryonated chicken eggs, were characterized biologically by conventional intracerebral pathogenicity indices (ICPI), and genetically on the basis of Phylogenic analysis of partial F-gene sequences (260 bp) encompassing the cleavage site. The ICPI values of isolates from chickens ranged from 0.9 to 1.8; whereas, the ICPI of pigeon isolates was 1.4. All isolates contained multiple basic amino acids at the deduced cleavage site of fusion protein, which is a typical feature of virulent viruses. Phylogenic analysis of the partial cleavage site of F-gene (260 bp) indicated that all the sequences of viruses obtained from pigeons were identical and clustered within the genotype VIh while the sequences of viruses obtained from chickens were clustered together within the genotype VIf. The similarity between the viruses obtained from chickens and those obtained from pigeons ranged from 82.5 to 85.6 %. This suggests that different sub genotypes of genotype VI are circulating in chicken and wild pigeon population in Ethiopia. This warrants further study to understand the role of wild birds in the epidemiology of NDV in Ethiopia and as well highlights the importance of continuous surveillances both in wild birds and domestic poultry.

## Background

Newcastle disease (ND) is a highly contagious and devastating disease of poultry caused by the Newcastle disease virus (NDV) also known as avian paramyxovirus type 1, (APMV-1). It is classified under the genus *Avulavirus* of family *Paramyxoviridae* (Mayo 2002). NDV is an enveloped nonsegmented, single-stranded, negative-sense RNA virus. Its genome has six open reading frames (ORF), which code for the following proteins: nucleoprotein (NP), phosphoprotein (P), matrix protein (M), fusion protein (F), hemagglutinin-neuraminidase (HN) and RNA-dependent RNA polymerase (L). During P-gene transcription, two additional nonstructural proteins, the V and W proteins are also generated through RNA editing (Steward et al. 1993).

Based on genomic size and the nucleotide sequences of F and L genes, NDVs can be categorized as class I or class II viruses. Class I NDVs are occasionally isolated from wild aquatic birds and domestic poultry and are mostly avirulent to chickens. Class II contains viruses that have been isolated from multiple wild birds and poultry species. Most viruses within this group are virulent and cause significant economic losses to poultry industry worldwide (Miller et al. 2009). These viruses are also highly diverse and continuously evolving (Czeglédi et al. 2006; Diel et al. 2011).

The key contributor to NDV pathogenicity is the formation of an active fusion protein upon cleavage of F protein precursor (F0) which is facilitated by the presence of a number of basic amino acid residues in fusion protein cleavage site (Toyoda et al. 1987; Glickman et al. 1988). NDVs that are virulent for chickens have a multiple basic amino acid sequence 112 R/K-R-Q-K/R-R 116 at C terminus of F2 protein and F (phenylalanine) at residue 117, which is the N-terminus of F1 protein, whereas the viruses of low virulence have a monobasic amino acid sequence in the same region of 112 G/E-K/R-Q-G/E 116 and L (leucine) at residue 117 (Kim et al. 2008).

NDV infects a wide range of domestic and wild bird species resulting in heavy economic losses to the poultry industry. In addition to poultry, more than 230 species from more than one-half of the 50 orders of birds have been shown to be susceptible to natural or experimental infections with avian paramyxovirus type 1 (Alexander and Senne 2008). Wild birds seem to be the reservoir of avirulent strains, whereas poultry are the most important host of the virulent viruses but both hosts exchange viruses (Cardenas et al. 2013). However, the identification of APMV-1 from birds showing clinical signs does not necessary imply the diagnosis of ND because of extreme variation in virulence of different APMV-1 isolates, co-infections and similar clinical signs caused by other diseases and widespread use of live vaccine. Thus, assessment of virulence of the isolates is required to confirm an ND outbreak (ICPI > 0.7, which is notifiable to the Office International Epizootes (OIE 2013).

In Ethiopia, ND is one of the most important diseases inflicting heavy losses of chickens (Chaka et al. 2012; Mazengia 2012). Despite this, the information about the genetic characteristics of the NDVs is limited to studies of a few samples collected from village chickens. Specifically, NDVs belonging to genotype VII (Fentie et al. 2013) and genotype VI (de Almeida et al. 2013; Chaka et al. 2013) were identified from village chickens in northwest and central Ethiopia, respectively. In addition, in our previous study (Delesa et al. 2014); we detected viruses of the subgenotype VIf, in village chickens in live bird markets. However, genetic profile of NDVs circulating in commercial farms is scarce and no information about NDVs circulating in wild birds in Ethiopia is available. To better understand the relationship between NDVs circulating in wild and domestic birds in this country, here we characterized NDV isolates obtained from ND outbreaks in different poultry farms and wild pigeons.

## Methods

### Sampling

**Fig. 1:**
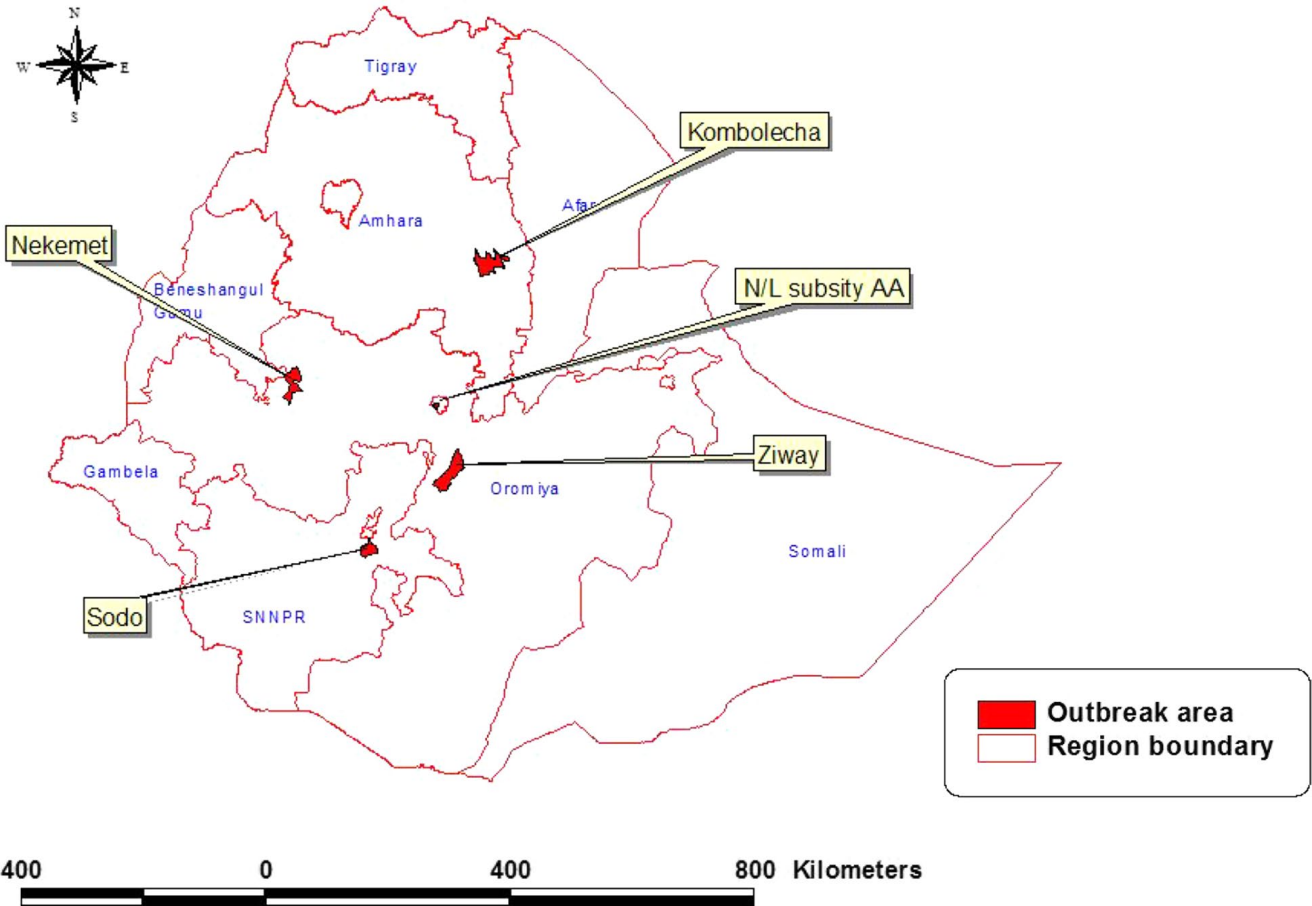
Location of outbreak areas where samples were collected

Between 2012 and 2014, a total of four suspected ND outbreaks in poultry farms and one outbreak in wild pigeons were investigated. Moribund birds or tissue samples of dead birds collected from suspected outbreaks were submitted to the National Animal Health Diagnostic Center (NAHDIC), Ethiopia. The affected farms were small scale poultry farms in Nekemte, Addis Ababa (Nefas silk), Sodo and Kombolcha districts (Fig. 1). The pigeon samples were collected from a suspected ND outbreak in wild pigeons around Lake Ziway. A total of eight isolates (two from each farm) obtained from chickens and four isolates obtained from four pigeons were characterized.

### Virus isolation

The selected organs (brain, trachea, lung and intestine) were collected from dead or humanely killed moribund birds, pooled and processed for virus isolation as indicated in the manual of the World Organization for Animal Health (OIE 2013). Briefly, 10 % w/v organ suspensions in viral transportation media (VTM) were homogenized, clarified and inoculated into the allantoic cavity of 9 to 11-day old embryonated chicken eggs (three eggs per sample) according to the standard virus isolation methods (Alexander and Senne 2008).

The inoculated eggs were candled every 24 h to check embryo vitality. Each egg containing a dead embryo on each day and all eggs at the end of incubation period were removed from incubator and chilled at +4 °C overnight. Allantoic fluids were harvested and tested by Haemagglutination (HA) test for its ability to haemagglutinate chicken RBCs. A minimum of two serial passages in embryonated eggs were performed for each sample to enhance viral copy number.

### Heamagglutination (HA) and haemagglutination inhibition (HI)

The HA and HI assays were carried out in microtiter plate as outlined by the World organization for Animal Health (OIE 2013). Inactivated NDV antigen (LaSota) strain was used as a positive control. Haemagglutinating agents were identified by means of the HI test using a monospesific antiserum against NDV raised in SPF chickens. Doubling dilutions of hyperimmune monospecific antiserum was used to inhibit the haemagglutinating activity of 4 HA units of the isolates. Isolates that showed inhibition of haemagglutination at the same serum dilution (±one dilution step) as the reference APMV-1 strain (LaSota) were typed as NDV. Isolates were further classified by means of the HI test using monoclonal antibody which is specific for pigeon paramyxoviruses (Alexander et al. 1997; Collins et al. 1989).

Inactivated NDV antigens (LaSota strain), monospesific antiserum against NDVs, monoclonal antibody against Pigeon paramyxoviruses and negative sera for HA/HI controls were from Istituto Zooprofilattico Sperimentale delle Venezie (OIE/FAO Reference Laboratory for AI and ND), Padua, Italy).

### Polymerase chain reaction

All HA positive allantoic fluids were analyzed by reverse transcription real—time PCR (RT-PCR) tests. Viral RNA extraction was conducted using Qiagen ^®^ Rneasy Mini kit according to manufacturer’s instruction. Real-time RT-PCR reactions were performed at the National Animal Health Diagnostic Center, using an applied Biosystem 7500 Fast Real-Time PCR thermo cycler. A primer-probe set targeting the F gene, Forward F + 4829 5′-GGT GAG TCT ATC CGG ARG ATACAA G-3′, Reverse Primer F- 4939 5′-AGC TGT TGCAAC CCC AAG-3′ and Probe F + 4894 5′-FAM-AAGCGT TTC TGT CTC CTT CCT CCA-BHQ-3′ (Wise et al. 2004) were used to amplify pathogenic strains of NDV. RT-PCR positive allantoic fluids were submitted to Istituto Zooprofilattico Sperimentale delle Venezie (OIE/FAO Reference Laboratory for AI and ND), Padua, Italy for further analyses.

### Pathogenicity of isolates

All isolates were tested at the National Anima Health Diagnostic and Investigation Center for virulence by intracerebral pathogenicity index (ICPI) test in day-old chickens according to (Alexander 1989).

### Sequence analysis of F protein cleavage site

To determine the amino acid sequence of the fusion protein cleavage site, the viral RNA was extracted directly from infected allantoic fluids for each isolate using the Nucleospin RNA II kit (Machery-Nagel, Duren Germany) according to the manufacturer’s instructions. Amplification was performed with primers NOH-For (5′-TAC ACC TCA TCC CAG ACA GG-3′) and NOH-Rev (5′-AGT CGG AGG ATG TGT TGG CAG C-3′) (Terregino and Capua 2009). The amplified DNA products were checked by electrophoresis through 1 % agarose gel and purified using ExoSAPIT (USB Corporation, Cleveland, OH). DNA products were subjected to direct nucleotide sequencing using v3.1 Cycle Sequencing Kit (Applied Biosystems, Foster city, CA, USA) and sequenced in a 16-capillary ABI PRISM 3130xl Genetic Analyzer (Applied Biosystem).

Pairwise sequence alignments were performed to determine nucleotide and amino acid sequence similarities. To determine the phylogenetic relationships, the 260-bp hypervariable region of the F gene sequence obtained in this study was compared with the corresponding regions of representative published class II NDV sequences available in GenBank. Alignment and comparison of the nucleotide and amino acid sequences were performed using Clustal W in MEGA 5.0 (Tamura et al. 2011). Maximum likelihood (ML) tree was estimated using the best-fit general time reversible (GTR) model of nucleotide substitution with gamma distributed rate variation among sites and a heuristic SPR branch—swapping search available in PhyML version 3.0 (Guindon and Gascuel 2003). A bootstrap resampling process (100 replicates) was employed to assess the robustness of individual nodes of phylogeny.

### Ethics approval

Sampling from animals were carried out according to the experimental practice and standards approved by the Animal Welfare and Research Ethics Committee at National Animal Health Diagnostic and Investigation Center (NAHDIC) that is in accordance with the international guidelines for animal welfare.

## Results

**Fig. 2:**
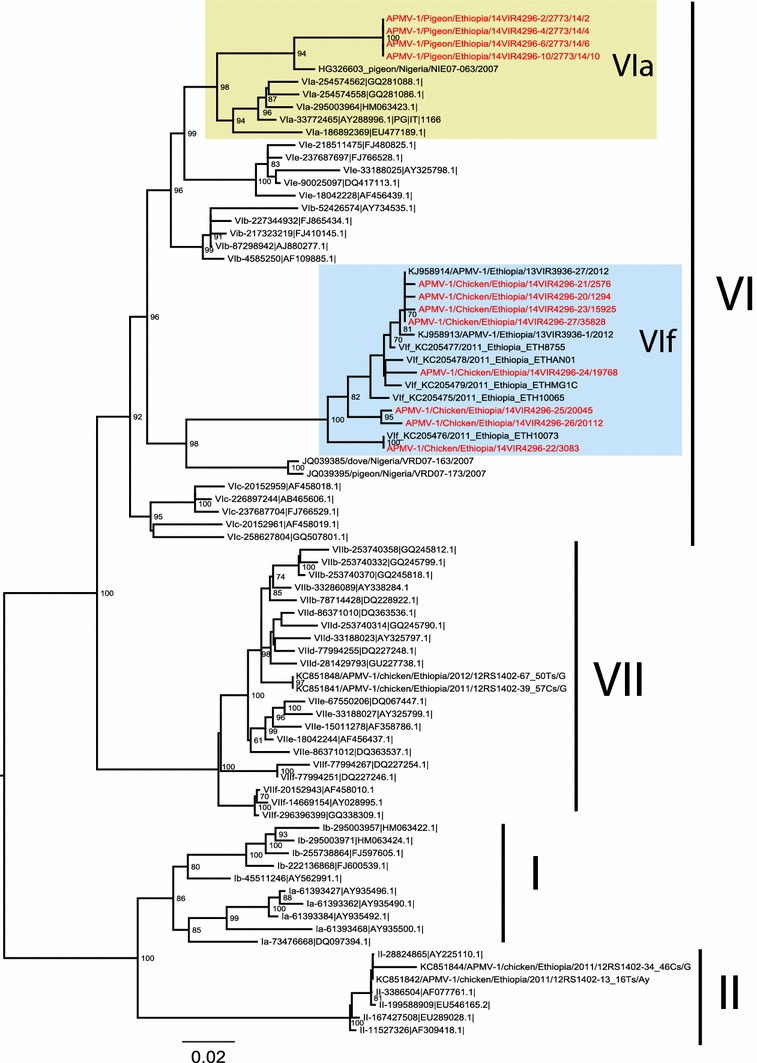
ML phylogenic analysis of partial F-gene sequences (260 bp) encompassing the cleavage site of APMV-1 viruses. Sequences under study are marked in *red*. *Numbers* at the nodes represent bootstrap values (>70)

All eight haemagglutinating isolates obtained from poultry farm outbreaks were identified as Newcastle disease virus that reacted with monospesific antiserum specific for avian paramyxovirus 1. All four isolates obtained from pigeon outbreak were identified as Pigeon paramyxovirus 1 that reacted with monoclonal antibody specific for Pigeon paramyxovirus 1. Besides, all the isolates were confirmed positive for virulent NDVs by reverse transcriptase real time PCR. The ICPI values of isolates from chickens ranged from 0.9 to 1.8; whereas, the ICPI of both pigeon isolates was 1.4 (Table 1). In addition, the pathotype prediction according to the deduced amino acid sequence of the cleavage site of the fusion protein showed that, the pigeon isolates have sequence motifs SVGRRRKR*F and chicken isolates have sequence motifs SGGRRQKR*F and SGGRRRKR*F suggestive of virulent strains (Table 1). Phylogenic analysis of partial F-gene sequences (260 bp) encompassing the cleavage site revealed that, all the sequences from pigeons (Fig. 2) were identical and clustered within the genotype VIh with viruses collected from pigeons in Nigeria between 2007 and 2013. The chicken viruses were clustered together with other Ethiopian isolates within the genotype VIf. However, isolates from the same farm do not always seem to end up in the same branch (Fig. 2). Specifically, one of the isolates from Kombolcha with sample Id 15925 is slightly different from the other isolate collected from the same place and was completely identical to the isolates from Sodo farm. It ended up on the same branch of the phylogenetic tree and contained the same cleavage site motifs (Table 1). The similarity between the viruses from chickens and those from pigeons ranged from 82.5 to 85.6 %.

**Table 1:** The ICPI and the sequence at fusion cleavage site of NDV isolates obtained from poultry farms and wild pigeons in Ethiopia

Isolate	Year of isolation	Species	Origin of isolate	ICPI	Cleavage site motif (112–117)	GenBank accession numbers
2773/14/2	2014	Pigeon	Ziway	1.4	RRRKR*F	KR014202
2773/14/4	2014	Pigeon	Ziway	1.4	RRRKR*F	KR014203
2773/14/6	2014	Pigeon	Ziway	nd	RRRKR*F	KR014204
2773/14/10	2014	Pigeon	Ziway	nd	RRRKR*F	KR014205
1294	2012	Chicken	Sodo	0.9	RRQKR*F	KR014206
2576	2012	Chicken	Sodo	nd	RRQKR*F	KR014207
3083	2014	Chicken	Kombolcha	1.2	RRRKR*F	KR014208
15925	2014	Chicken	Kombolcha	nd	RRQKR*F	KR014209
19768	2013	Chicken	Nekemte	1.5	RRQKR*F	KR014210
20045	2013	Chicken	Nekemte	nd	RRQKR*F	KR014211
20112	2013	Chicken	Addis ababa	1.8	RRQKR*F	KR014212
35828	2013	Chicken	Addis ababa	nd	RRQKR*F	KR014213

*nd* not done

## Discussions

In this study, we characterized NDV isolates that were obtained from suspected outbreaks of ND in small scale poultry farms and in wild pigeons between 2012 and 2014 in Ethiopia. In vivo analysis showed that all the isolates were virulent with ICPI ranging from 0.9 to 1.8. In addition, all the isolates contained multiple basic amino acids at the deduced cleavage site of fusion protein, which is a typical feature of virulent viruses. Virulent NDV is an APMV-1 with an ICPI of 0.7 or higher or with at least three basic amino acids between positions 113 and 116 and phenylalanine at position 117 (Aldous and Alexander 2001). The present findings are in agreement with earlier reports that revealed the circulation of virulent NDVs in the village chickens in Ethiopia (Delesa et al. 2014; de Almeida et al. 2013; Chaka et al. 2012).

Phylogenic analysis of the 260 fragment of the fusion gene of all eight isolates obtained from commercial poultry farms showed that, the viruses were classified under the new subgenotype VIf of class II viruses, and grouped with NDVs previously identified from village chickens in Ethiopia (Delesa et al. 2014; de Almeida et al. 2013; Chaka et al. 2013) regardless of the geographical origin of the isolates. This suggests circulation of the same genotype within the poultry farms. However, one of the isolates from Kombolcha (15925) collected in 2014 was slightly different from the other isolate from the same place and completely identical to isolate from Sodo (2576) collected in 2012; ended up on the same branch of the phylogenetic tree and contained the same cleavage site motifs. This suggests the presence of mixed population of NDVs in these farms. Besides, the circulation of identical NDVs in both farms indicates the continuous movement of the viruses in between poultry farms situated in different parts of the country. However, this needs further investigations since the number of isolates was very small in this study.

The close similarity of the NDVs from commercial small scale poultry farms with those from village chickens suggests that viruses can move reasonably freely between these two bird populations. This could be attributed to the poor biosecurity practices in these farms and live poultry markets in Ethiopia. In live poultry markets birds from different sources (villages and small scale commercial farms) are mixed and sold either to traders or to the consumers. Unsold birds are usually taken back to their respective farms and villages (Shewantasew et al. 2012; Delesa et al. 2014).

Despite routine vaccination with Hitchner B1 (HB1) and LaSota strains, frequent ND outbreaks occur in small scale commercial flocks. These vaccine strains belong phylogenetically to the same genotypes (I and II) and are divergent from NDVs that caused the current outbreaks. ND outbreaks could occur despite intensive vaccination against NDV (Bogoyavlenskiy et al. 2009; Hassan et al. 2010; Ke et al. 2010). This might be associated with genetic and antigenic divergence between the vaccine strain and the circulating field strains (Hu et al. 2009; Miller et al. 2009). However, poor vaccine application or poor quality of the vaccines used can’t be excluded and hence, further investigation is required to understand the root causes of vaccine failures.

To our knowledge, this is the first study to genetically characterize NDVs circulating in pigeons in Ethiopia. All four isolates obtained from pigeons were NDVs with ICPI ranging from 1.2 to 1.4 and contained multiple basic amino acids at the deduced cleavage site of fusion proteins. Phylogenetic analysis of the isolates showed that the viruses belonged to the new sub genotype VIh (Snoeck et al. 2013) and were closely related to isolates obtained from pigeons in Nigeria and UAE. The finding is consistent with the epidemiology of Genotype VI viruses which affect multiple avian species including doves and pigeons (Kim et al. 2008).

The circulation of genotype VI in both domestic poultry and wild pigeons in Ethiopia provide strong evidence that wild birds may play roles in epidemiology of NDVs in this country. Specially, village chickens that represent 97 % of the national flock (CSA 2012), operate in an environment exposed to contamination by migrating and resident wild birds and are at high risk of acquiring the disease. But this needs to be further investigated to understand the role of wild birds in the epidemiology of ND in domestic birds. Similar findings were reported from western Africa where virulent NDVs, including genotype VIh and genotype XVII were identified from wild birds (Snoeck et al. 2013).

## Conclusion

ND represents a significant ongoing threat to domestic and wild bird populations in Ethiopia. Analysis of the eight isolates from small scale commercial poultry farms and four isolates from wild pigeon outbreaks indicated the circulations of virulent NDVs that belong to genotype VIf and VIh in chickens and pigeons respectively. This study provides valuable information on epidemiology of NDVs circulating in the country and thus highlights the importance of continuous surveillance of ND both in commercial poultry farms and in wild birds. Besides, the role of wild birds in epidemiology of ND and efficacy of current ND vaccines and vaccination practices should be investigated so that coordinated control strategies can be established.
